# Endovascular Treatment of Ruptured Wide-Necked Anterior Communicating Artery Aneurysms Using a Low-Profile Visualized Intraluminal Support (LVIS) Device

**DOI:** 10.3389/fneur.2020.611875

**Published:** 2021-01-28

**Authors:** Gaici Xue, Peng Liu, Fengfeng Xu, Yibin Fang, Qiang Li, Bo Hong, Yi Xu, Jianmin Liu, Qinghai Huang

**Affiliations:** ^1^Department of Neurosurgery, General Hospital of Southern Theatre Command of People's Liberation Army of China, Guangzhou, China; ^2^Department of Neurosurgery, Changhai Hospital, Navy Medical University, Shanghai, China; ^3^Department of Neurosurgery, Naval Medical Center of People's Liberation Army of China, Navy Medical University, Shanghai, China

**Keywords:** intracranial aneurysm, ruptured, anterior communicating artery, LVIS stents, safety, wide-necked aneurysms

## Abstract

**Objective:** To evaluate the safety and efficacy of low-profile visualized intraluminal support (LVIS) stent-assisted coiling for the treatment of ruptured wide-necked anterior communicating artery (ACoA) aneurysms.

**Methods:** The clinical and angiographic data of 31 acutely ruptured wide-necked ACoA aneurysms treated with LVIS stent-assisted coiling between January 2014 and December 2018 were retrospectively reviewed.

**Results:** All stents were successfully deployed. The immediate angiographic results were modified Raymond-Roy class I in 27 cases, modified Raymond-Roy class II in 2 cases, and modified Raymond-Roy class IIIa in 2 cases. Intraoperative thrombosis and postoperative aneurysmal rebleeding occurred in one case each. Two patients (6.5%) who were admitted due to poor clinical grade conditions died during hospital admission as a result of initial bleeding. Angiographic follow-up (mean: 12.9 months) was performed for 26 patients, the results of which demonstrated that 25 aneurysms were completely occluded and one was class II. The last clinical follow-up (mean: 25.3 months) outcomes demonstrated that 27 patients had favorable clinical outcomes and two had poor clinical outcomes.

**Conclusion:** LVIS stent-assisted coiling for ruptured wide-necked ACoA aneurysms was safe and effective, with a relatively low rate of perioperative complications and a high rate of complete occlusion at follow-up.

## Introduction

The anterior communicating artery (ACoA) is one of the most common sites of ruptured intracranial aneurysms (RIAs) (1, 2). Endovascular treatment has become an important approach to manage these lesions (3–7). However, endovascular coiling of ruptured wide-necked ACoA aneurysms is still technically challenging because of the small vessel diameter, very small size, and accompanying hematoma. Our previous studies have shown that stent-assisted coiling achieved favorable outcomes in the treatment of ruptured wide-necked ACoA aneurysms (8, 9). Nevertheless, compared with a non-braided stent, whether the low-profile visualized intraluminal support (LVIS) device (MicroVention, Tustin, CA, USA), which is unsuitable for the Y-stent technique due to a small mesh, can be fully opened in the small-diameter vessel of the anterior communicating complex, and whether it can provide good protection to incorporating vessels is not well-reported. Therefore, we herein present a patient cohort with ruptured wide-necked ACoA aneurysms treated with LVIS stent-assisted coiling, in which we evaluated the safety and efficacy of this strategy.

## Materials and Methods

The local Institutional Review Board approved the present study protocol. The requirement for written informed consent was waived due to the retrospective nature of the study.

### Patient Population and Selection

Between January 2014 and December 2018, 328 patients with 328 ruptured ACoA aneurysms were admitted to our institution. We excluded (a) aneurysms treated more than 3 days after the initial rupture; (b) aneurysms with traumatic, infectious, pseudo, fusiform, dissecting, and blood blister-like properties; (c) aneurysms treated by surgical clipping; and (d) those combined with other severe cerebral diseases (Arteriovenous Malformation, moyamoya, Dural arteriovenous fistulae, Carotid-Cavernous Fistula, tumor) that also required treatment. Finally, 31 patients with 31 ruptured wide-necked ACoA saccular aneurysms treated with LVIS stent-assisted coiling, and 241 ruptured ACoA aneurysms treated with other therapies, including 12 cases with laser-cut stent-assisted coiling, 34 with balloon-assisted coiling, and 195 with simple coiling, were included.

### Endovascular Procedure

All procedures were performed with the patients under general anesthesia. A 6F guiding catheter was introduced through the femoral artery and placed in the distal internal carotid artery. Three-dimensional (3D) reconstructions of rotational digital subtraction angiography (DSA) were performed to obtain the morphology and size of the aneurysm and parent artery. Optimal working projections were obtained for coil packing and stent delivery after adequate assessment of the aneurysm size and dome projection, as well as the angle between the A1 and A2 segments and the tortuosity of the involved vasculature. The stent microcatheter (Headway 21; MicroVention, Tustin, California, USA) was delivered to the contralateral A2 or ipsilateral A2 segment of the anterior cerebral artery for stent deployment as appropriate. The Echelon-10 (Covidien/ev3; Irvine, California, USA) or Headway 17 (MicroVention, Tustin, California, USA) was navigated into the aneurysm sac. After the first coil was introduced, the stent was deployed and completely covered the aneurysm neck using the semi-jailing technique to ensure that the stent could be fully opened and apposed well across the aneurysm neck. The aneurysm was packed with more coils until aneurysm obliteration was considered adequate. For the cases scheduled for coiling only, bailout stenting was used to compress the coil into the aneurysm sac when the coil protruded into the parent artery.

### Antiplatelet and Anticoagulation Therapy

Throughout the procedure, systemic heparinization was performed for all patients with an activated clotting time of 2–3 times that of the baseline. A loading dose of aspirin (300 mg) and clopidogrel (300 mg) was administered rectally immediately after the operator decided to deploy a stent. Glycoprotein IIb/IIIa inhibitor (tirofiban; Grand Pharma, Wuhan, China) was administered (5 μg/kg for 3 min) intravenously before stent deployment and was maintained at a rate of 0.075 μg/kg/min until 6 h after the loading dose of aspirin and clopidogrel. In the postoperative period, all patients continued to receive aspirin (100 mg daily) and clopidogrel (75 mg daily) for 6 weeks, followed by 100 mg of aspirin daily alone indefinitely.

### Clinical and Angiographic Evaluation

All surviving patients were followed up through clinical evaluation or telephone interview at 3, 6, and 12 months, and once a year thereafter. The clinical outcomes at discharge and follow-up were evaluated using the modified Rankin Scale (mRS). Favorable clinical outcomes were defined as an mRS score of 0–2, and poor clinical outcomes were defined as an mRS score of 3–6.

The immediate embolization results and angiographic follow-up results were evaluated according to the modified Raymond-Roy classification. Postoperative angiographic follow-up was recommended for all surviving patients, including 3-month magnetic resonance angiography (MRA), 6-month DSA, and MRA or DSA yearly thereafter (10).

## Results

### Patient Enrollment and Baseline Characteristics

Of the 31 patients, 12 (38.7%) were men and 19 (61.3%) were women. The patients' age ranged between 35 and 85 years (mean: 55.9 years). There were 19 (61.3%) aneurysms with superior projection, eight (25.8%) with inferior projection, three (9.7%) with anterior projection, and one (3.2%) with complex projection. Dysplasia of one A1 segment was encountered in nine (29.0%) patients. The aneurysm neck involved the ipsilateral A2 in 13 (41.9%) cases, the contralateral A1 in seven (22.6%), and bilateral A2 in 11 (35.5%).

### Immediate Embolization Results and Peri-Procedure Complications

**Figure 1:**
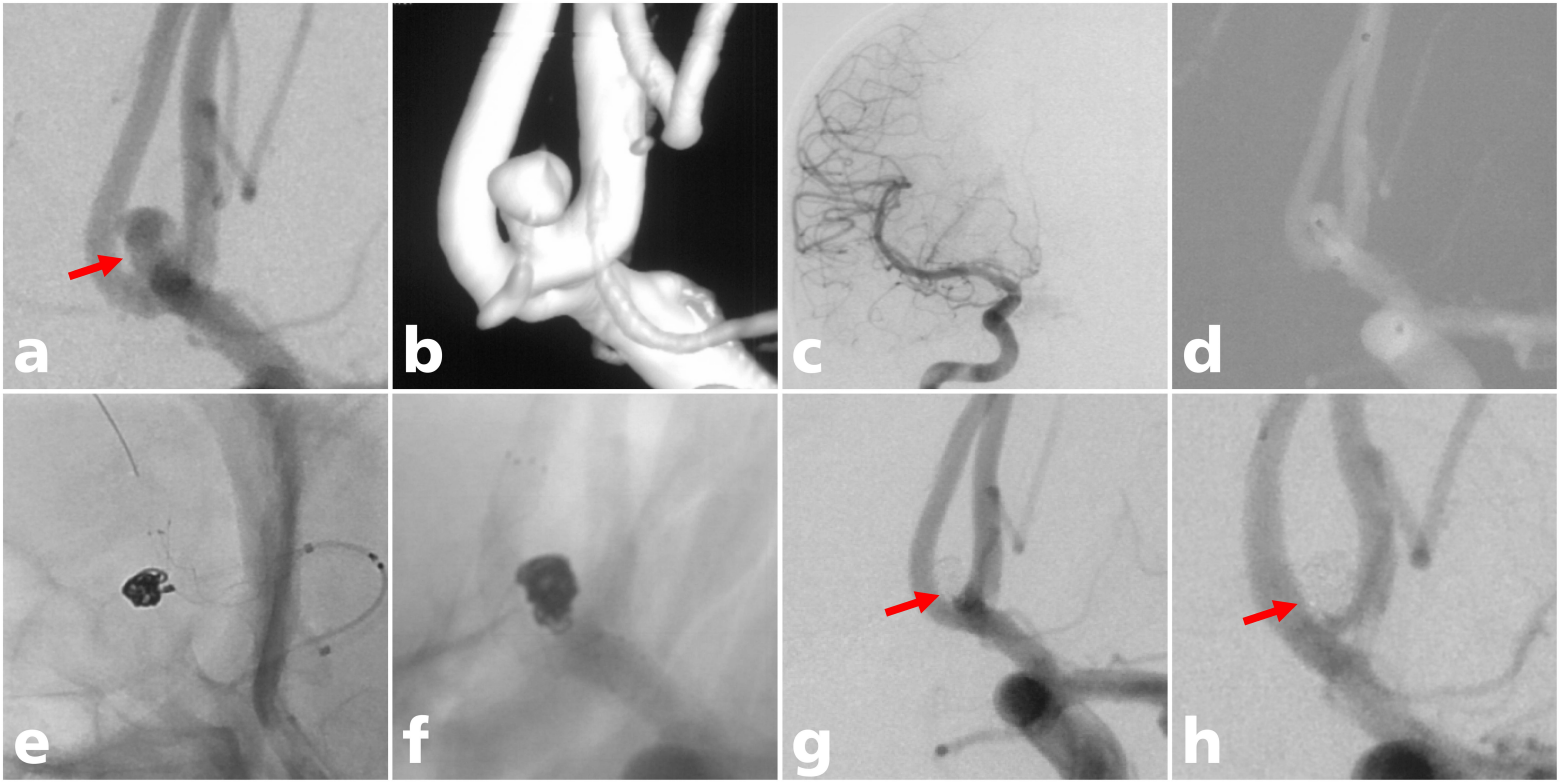
A 38-year-old man with an ACoA aneurysm treated with LVIS stent-assisted coiling. **(a)** A wide-necked ACoA aneurysm in which the aneurysm neck mostly involved the ACoA (solid arrow); **(b)** three-dimensional reconstruction of the aneurysm; **(c)** aplasia of the right A1 segment; **(d)** the microcatheter was delivered into the aneurysm sac to place the coils; **(e)** the stent microcatheter was exchanged to the contralateral A2 segment for stent deployment; **(f)** the LVIS stent (3.5 mm × 15 mm) was successfully deployed using the semi-jailing technique; **(g)** complete occlusion was achieved under final view (solid arrow); **(h)** complete occlusion (modified Raymond-Roy class I) of the aneurysm at 12-month angiographic follow-up (solid arrow).

**Figure 2:**
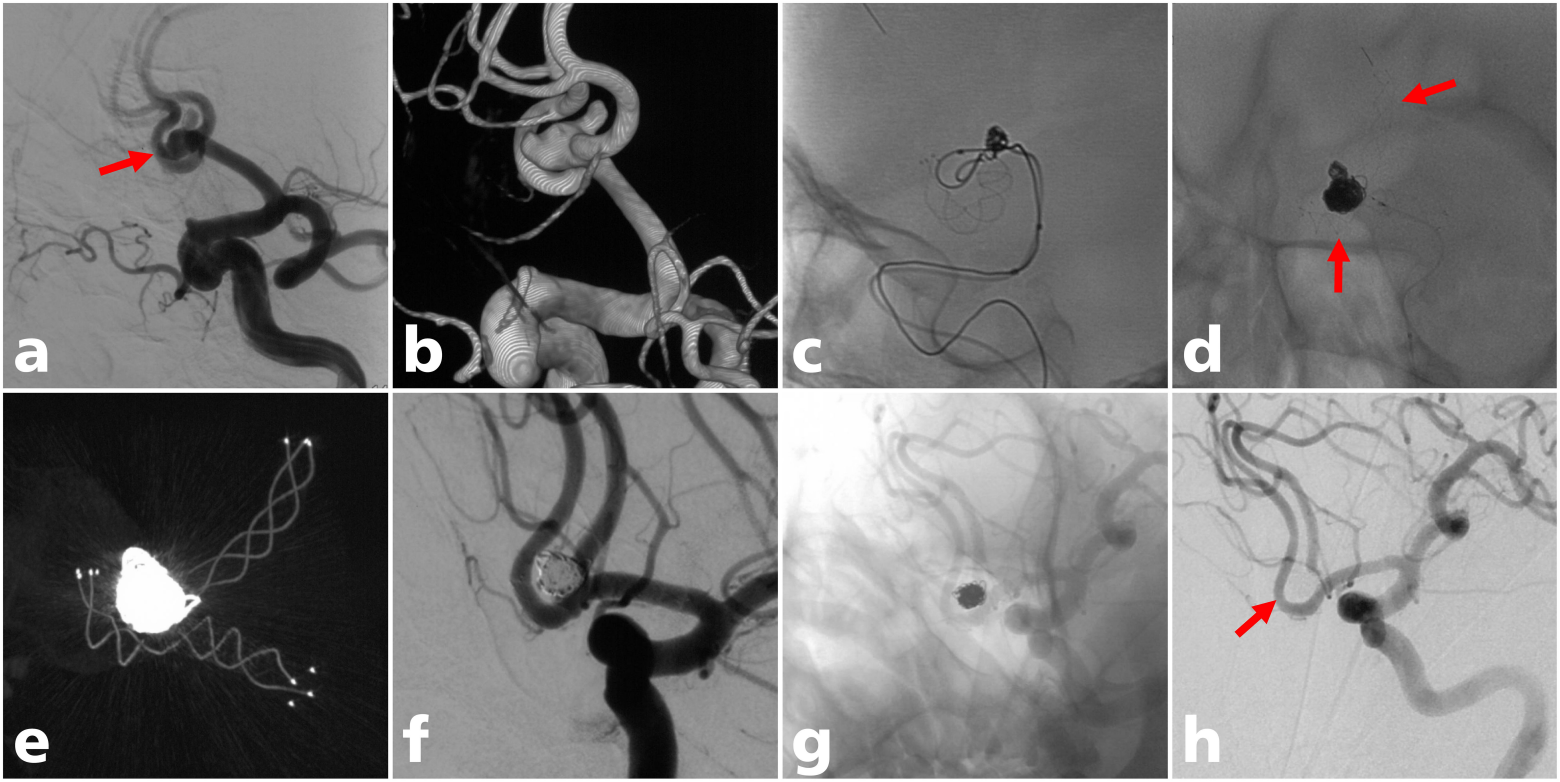
A 60-year-old man with an ACoA aneurysm treated with LVIS stent-assisted coiling with a T configuration. **(a)** Angiogram showed a wide-neck ACoA aneurysm (solid arrow); **(b)** three-dimensional reconstruction of the aneurysm; **(c)** the LVIS stent (3.5 mm × 20 mm) was successfully deployed crossing the ACoA to the contralateral A2 segment; **(d)** the LVIS Jr stent (3.5 mm × 18 mm) was successfully deployed from the ipsilateral A2 to the A1 segment in a T configuration (solid arrow); **(e)** postoperative CT reconstruction showed that the two stents were complete opened **(f)** partial occlusion (modified Raymond-Roy class IIIa) was achieved under final view; **(g,h)** complete occlusion (modified Raymond-Roy class I) of the aneurysm at last angiographic follow-up (solid arrow).

All LVIS stents were successfully implanted for the 31 ruptured wide-necked ACoA aneurysms (illustrative case shown in Figure 1). No stent displacement or migration occurred during the procedures. Bailout stenting was performed in two cases (6.5%). Immediate embolization results demonstrated that modified Raymond-Roy class I was achieved in 27 (87.1%) cases, modified Raymond class II in 2 (6.5%), and modified Raymond class IIIa in 2 cases (6.5%). T-configuration stent deployment was performed in two (6.5%) cases for bilateral A2 protection (illustrative case shown in Figure 2). The stent was positioned across the ACoA from A1 to the contralateral A2 segment in 18 (58.1%) patients and to the ipsilateral A2 segment in 11 (35.5%) patients.

Intraoperative thrombosis was observed in one patient. When more coils were placed into the aneurysm sac to achieve greater aneurysm packing, one coil protruded into the parent artery and acute thrombosis occurred around the coil loop. A loading dose (10 μg/kg for 3 min) of tirofiban was immediately administered proximal to the thrombus intra-arterially through the microcatheter; this allowed the operator to confirm that the blood flow was restored, and a maintenance dose of tirofiban (0.075 μg/kg/min) was subsequently administered intravenously for 6 h. Next, a Headway 21 microcatheter was navigated across the ACoA to the contralateral A2 segment, and as a bailout technique, an LVIS stent (3.5 mm × 15 mm) was deployed, covering the aneurysm neck to press the coil into the aneurysm sac. Fortunately, the thrombus disappeared quickly, and no neurological deficit occurred in this patient after the endovascular procedure. Postoperative aneurysmal rebleeding occurred in one patient; in this patient, the LVIS stent was deployed successfully and no complications were observed during the entire endovascular procedure. Immediate embolization results demonstrated that modified Raymond-Roy class I was achieved. However, the patient suffered cerebral hernia 1 day after the endovascular procedure. The computed tomography scan showed an increased hematoma in the right frontal lobe, and aneurysmal rebleeding was suspected. Decompressive craniotomy, right frontal lobe hematoma evacuation, and ACoA aneurysm wrapping were performed. The patient was dependent on an mRS score of 3 at discharge and recovered well with an mRS score of 1 at the last clinical follow-up.

### Clinical Outcomes

At discharge, 26 (83.9%) patients were independent, with an mRS score of 0–2, three (9.7%) patients were dependent on an mRS score of 3–5, and two (6.5%) patients died due to severe cerebral vasospasm. The results of the last clinical follow-up (mean: 25.3 months) showed that 27 patients (93.1%) had favorable clinical outcomes, while the remaining two (6.9%) had poor clinical outcomes.

### Angiographic Follow-Up Results

Angiographic follow-up (mean: 12.9 months) was performed for 26 patients, and the results indicated that 25 (96.1%, 25/26) aneurysms were completely occluded and one (3.9%, 1/26) was stable. No in-stent stenosis or parent artery occlusion were observed.

### Outcomes for Other Therapies

With regard to the three other therapies, laser-cut stent-assisted coiling achieved modified Raymond-Roy class I in 10 (83.3%), while two (16.7%) developed perioperative complications, including one intraoperative rupture and one intraoperative thrombosis. In addition, 9/10 (90%) patients showed complete long-term occlusion, 34 patients had accepted balloon-assisted coiling, and 26 (76.5%) patients achieved immediate complete occlusion. Two patients developed perioperative complications. Of the 20 patients who underwent long-term follow-up, 17 achieved complete occlusion. Simple coiling was administered to 195 patients, resulting in 137 (70.3%) immediate complete occlusions and 12 complications. Long-term imaging follow-up showed 112 (79.4%) occlusions in 141 patients (Table 1).

**Table 1:** Results of different methods used for the treatment of ruptured ACoA aneurysms.

	**LVIS stent-assisted coiling**	**Laser-cut stent-assisted coiling**	**Balloon-assisted coiling**	**Simple coiling**
Number of patients	31	12	34	195
Immediate complete occlusion rate	27/31 (87.1%)	10/12 (83.3%)	26/34 (76.5%)	137/195 (70.3%)
Perioperative complication rate	2/31 (6.5%)	2/12 (16.7%)^*^^1^	2/34 (5.9%)^*^^2^	12/195 (6.2%) ^*^^3^
Long-term occlusion rate	25/26 (96.1%)	9/10 (90%)	17/20 (85.0%)	112/141(79.4%)

*^1^ *One case of intraoperative rupture and one case of intraoperative thrombosis*.

*^2^ *One case of postoperative aneurysmal rebleeding and one case of intraoperative thrombosis*.

*^3^ *Eight cases of intraoperative thrombosis, three cases of intraoperative aneurysm rupture, and one case of postoperative aneurysmal rebleeding*.

## Discussion

This study is the first report of LVIS stent-assisted coiling for acutely ruptured wide-necked ACoA aneurysms. The results demonstrate procedure-related morbidity and mortality of 6.5% and 0, respectively, and 93.1% of the patients had favorable clinical outcomes at follow-up. The complete occlusion rate immediately after the procedure was 87.1%, which increased to 96.1% at the last angiographic follow-up. These results suggest that LVIS stent-assisted coiling for acutely ruptured wide-necked ACoA aneurysms could result in favorable clinical and angiographic outcomes, with a relatively low rate of procedure-related complications.

Stent-assisted coiling for intracranial wide-necked aneurysms has been well-documented. Stenting strategies, including the crossing technique, stenting after coiling (11), semi-jailing (12), Y-configuration (13), and T-configuration (14), vary according to the branch vessels involved in the aneurysm neck. As for ACoA, whether the contralateral A1 is dysplastic is an important factor to be considered when establishing the strategy for positioning the stent (15). For patients with dysplasia or no contralateral A1 segment, the stent was generally positioned crossing the ACoA from one A1 segment to the contralateral A2 segment to protect the patency of the ACoA and branch vessels. For patients without dysplasia of the contralateral A1 segment and in those without contralateral A2 involvement, the stent was regularly positioned from the ipsilateral A2 to the A1 segment. For patients in whom the bilateral A2 segment was involved in the aneurysm neck, the repeated “pull-push” technique was used to make the stent partially protruding into the aneurysm to provide better protection to the involved vessels. The LVIS stent has a small mesh (<1 mm), especially when placed into a small-diameter vessel; thus, a second stent deployment with Y-configuration crossing the small mesh was difficult, as was complete opening and good apposition to the vessel wall. Therefore, the Y-configuration technique is considered unsuitable for LVIS stents. However, for patients with an extremely wide aneurysm neck, it may be necessary to use the T-configuration technique to implant a stent with smaller outer diameters, such as a LVIS Junior stent (LVIS Jr), in the other vessel.

The ACoA complex often involves small-diameter vessels and a sharp angle between the A1 and A2 segments. One concern following stenting in such vessels is the risk of incomplete stent expansion, which is a common cause of periprocedural thromboembolic complications (16). Earlier studies have demonstrated that the LVIS stent has a higher likelihood of incomplete stent expansion in the tortuous parent arteries or the acute angle between the parent and daughter artery due to its low radical force (16, 17). Cho et al. reported five cases (18.5%, 5/27) of segmentally incomplete stent expansion where the LVIS stent was deployed from the distal internal carotid artery to the cavernous internal carotid artery (17). However, no incomplete stent expansion or stent migration events were observed in the present study, which may be due to greater operator experience. More importantly, the internal carotid artery was fixed within the rigid bone structure from the petrous segment to the paraclinoid segment, which is a limitation for stent deployment. In contrast, the ACoA is relatively free within the subarachnoid space, and stent placement can significantly change the angle between efferent and afferent vessels, which facilitates complete opening of the stent and better apposition to the vessel wall (18). All of the stents in our study were released successfully before the packing was complete. In addition, a cone-beam CT scan was used to ensure that the stent was fully opened and well-apposed.

The periprocedural complication rates of ACoA aneurysms treated with non-LVIS stent-assisted coiling range from 0 to 34.6% (9, 19–22). Fan et al. reported 63 patients with ruptured ACoA aneurysms who underwent laser-cutting stent-assisted coiling, and found that the rates of intraprocedural aneurysm rupture and thrombus formation were 9.5% (6/63) and 15.9% (10/63), respectively (19). Similarly, Yang et al. retrospectively reviewed 45 cases of ruptured ACoA aneurysms treated with non-LVIS stent-assisted coiling, and the results revealed that the rates of ischemic complications and intraoperative rupture were 17.8% (8/45) and 8.9% (4/45), respectively (20). Our previous study, with 27 cases of ruptured wide-necked ACoA aneurysms treated with Enterprise stent-assisted coiling, demonstrated a procedure-related complication rate of 7.4% (2/27) (9). However, LVIS stents have also been reported to have a high perioperative thromboembolic complication rate due to their high metal coverage, which ranges from 3.5 to 4.9% (16, 23, 24). A recent meta-analysis suggested that the most common periprocedural complications of LVIS stents for the treatment of intracranial aneurysms were thromboembolisms and in-stent thrombosis (23). Similarly, Mokin et al. compared the differences between Enterprise stents, Neuroform stents, and LVIS stents in the treatment of intracranial aneurysms, and found that procedure-related complications were higher in the LVIS stents than in the Enterprise and Neuroform stent (24). Although there has been no direct comparison of different types of stents, the results of our study, with a procedure-related complication rate of 6.5%, seem favorable. The lower complication rate may be related to the braided characteristic; the LVIS stent can fully expand at the neck of the aneurysm to protect the neck and vessels. Furthermore, the use of cone-beam CT can help make an accurate judgment about whether the LVIS stent has effectually opened and incorporated vessels. This will significantly reduce complications of thromboembolism. With a small mesh (<1 mm), LVIS can effectively prevent coils from protruding into the vessel; this enables the selection of a smaller coil to make the procedure safer and significantly reduces the probability of aneurysm perforation.

Stenting in ruptured intracranial aneurysms remains controversial because of the need for antiplatelet medication, which carries the risk of theoretically elevating aneurysm rebleeding and surgery-related hemorrhage for patients requiring surgery. The endosaccular flow disruption device [WEB (Woven EndoBridge), LUNA AES (LUNA Aneurysm Embolization System), MED (Medina Embolic Device), and Contour (Contour Neurovascular System)] were designed to target the aneurysm neck and block the blood flow into the aneurysm, thus promoting intratumoral thrombosis and neo-endothelialization at the aneurysm neck (25). Theoretically, an endosaccular flow disruption device is an ideal treatment option for ruptured intracranial aneurysms because antiplatelet medication is not required. Of these endosaccular flow disruption devices, the WEB is the most widely used in clinical practice, while the other three devices are rarely reported for the treatment of ruptured intracranial aneurysms (26). To date, no studies have specifically evaluated the safety and efficacy of WEB for the treatment of ruptured ACoA aneurysms. A systematic review by van Rooij et al. showed that for patients with ruptured intracranial aneurysms treated with WEB, the procedure-related complication rate ranged from 0 to 27.3%, the thromboembolic complication rate ranged from 9.4 to 21%, the procedure-related mortality ranged from 1.0 to 12.1%, and the follow-up aneurysm occlusion rate ranged from 33.3 to 73.0% (27). Some clinicians initiate antiplatelet medication for patients with ruptured intracranial aneurysms treated with WEB given the high incidence of thromboembolic complications. Nevertheless, the dose and duration of antiplatelet therapy varies widely in different studies (27). Moreover, there remain concerns that the WEB devices can protrude into the parent artery in some more complex cases, and carry the risk of thromboembolic events; this requires the use of salvage stent placement to stabilize the WEB into the aneurysm sac (28). Indeed, a retrospective study reported by Maurer et al. (29) showed that the incidence of protrusion of WEB into the parent artery was 4%. Overall, the safety and long-term efficacy of WEB in the treatment of ruptured intracranial aneurysms still need to be further assessed.

The pCONus is a new neck remodeling device designed for bifurcated wide-necked aneurysms. Drug-coated stents designed to have a tissue organization effect on the outer aneurysmal side of the stent and an antithrombotic effect on the inner stent blood-facing side might be a promising device to treat ruptured intracranial aneurysms. Daisuke et al. conducted an *in vitro* experiment on a drug-containing stent capable of controlled release of basic fibroblast growth factor and argatroban to treat rabbit cerebral aneurysms. The results showed that the majority of the aneurysm cavity was occupied by loose connective tissues in the drug-coated stent group, whereas extensive massive hematomas were observed in the drug-free stent group. Moreover, the drug-coated stents had a relatively low rate of in-stent thrombosis (30). However, the feasibility and safety of drug-coated stents for the treatment of human intracranial aneurysms still needs to be further explored.

The retrospective design has several limitations. First, the lack of a control group is likely the major limitation of the study. Second, this case series had a small sample size and all patients were from only one institution, which may have resulted in selection bias. Third, the self-adjudication of clinical and angiographic outcomes is bias that is inherent to this type of study. However, this is the first study to report the safety and efficacy of LVIS stent-assisted coiling for the treatment of acutely ruptured wide-necked ACoA aneurysms. Our findings may provide an alternative treatment option for ruptured wide-necked ACoA aneurysms.

## Conclusions

The LVIS stent-assisted technique is a feasible, safe, and promising option for the treatment of acutely ruptured wide-necked ACoA aneurysms. Further follow-up is needed to adequately assess the long-term efficacy of this strategy.

## Data Availability Statement

The raw data supporting the conclusions of this article will be made available by the authors, without undue reservation.

## Ethics Statement

Ethical review and approval was not required for the study on human participants in accordance with the local legislation and institutional requirements. The patients/participants provided their written informed consent to participate in this study.

## Author Contributions

QH, BH, YX, and JL participated in the design of this study. GX, PL, and FX drafted the manuscript. PL and FX performed statistical analysis. GX carried out the collected important background information. YF and QL carried out literature search. QH modified the manuscript. All authors read and approved the final manuscript.

## Conflict of Interest

The authors declare that the research was conducted in the absence of any commercial or financial relationships that could be construed as a potential conflict of interest.
